# Citrus Genetic Transformation: An Overview of the Current Strategies and Insights on the New Emerging Technologies

**DOI:** 10.3389/fpls.2021.768197

**Published:** 2021-11-30

**Authors:** Gabriela Conti, Beatriz Xoconostle-Cázares, Gabriel Marcelino-Pérez, Horacio Esteban Hopp, Carina A. Reyes

**Affiliations:** ^1^Instituto de Agrobiotecnología y Biología Molecular, UEDD INTA-CONICET, Hurlingham, Argentina; ^2^Cátedra de Genética, Universidad de Buenos Aires, Buenos Aires, Argentina; ^3^Departamento de Biotecnología y Bioingeniería, Centro de Investigación y de Estudios Avanzados del Instituto Politécnico Nacional, Mexico City, Mexico; ^4^Laboratorio de Agrobiotecnología, Facultad de Ciencias Exactas y Naturales, Departamento de Fisiología, Biología Molecular y Celular (FBMC), Universidad de Buenos Aires, Buenos Aires, Argentina; ^5^Instituto de Biotecnología y Biología Molecular, CCT-La Plata, CONICET-UNLP, Buenos Aires, Argentina

**Keywords:** citrus transgenic plants, *in vitro* regeneration, transformation methods, CRISPR in citrus, reporter and selection markers, cisgenesis and intragenesis, citrus promoters, citrus biotechnology

## Abstract

Citrus are among the most prevailing fruit crops produced worldwide. The implementation of effective and reliable breeding programs is essential for coping with the increasing demands of satisfactory yield and quality of the fruit as well as to deal with the negative impact of fast-spreading diseases. Conventional methods are time-consuming and of difficult application because of inherent factors of citrus biology, such as their prolonged juvenile period and a complex reproductive stage, sometimes presenting infertility, self-incompatibility, parthenocarpy, or polyembryony. Moreover, certain desirable traits are absent from cultivated or wild citrus genotypes. All these features are challenging for the incorporation of the desirable traits. In this regard, genetic engineering technologies offer a series of alternative approaches that allow overcoming the difficulties of conventional breeding programs. This review gives a detailed overview of the currently used strategies for the development of genetically modified citrus. We describe different aspects regarding genotype varieties used, including elite cultivars or extensively used scions and rootstocks. Furthermore, we discuss technical aspects of citrus genetic transformation procedures *via Agrobacterium*, regular physical methods, and magnetofection. Finally, we describe the selection of explants considering young and mature tissues, protoplast isolation, etc. We also address current protocols and novel approaches for improving the *in vitro* regeneration process, which is an important bottleneck for citrus genetic transformation. This review also explores alternative emerging transformation strategies applied to citrus species such as transient and tissue localized transformation. New breeding technologies, including cisgenesis, intragenesis, and genome editing by clustered regularly interspaced short palindromic repeats (CRISPR), are also discussed. Other relevant aspects comprising new promoters and reporter genes, marker-free systems, and strategies for induction of early flowering, are also addressed. We provided a future perspective on the use of current and new technologies in citrus and its potential impact on regulatory processes.

## Introduction

The genus *Citrus* of the Rutaceae family is one of the most important commercial woody fruit crops from tropical and subtropical areas of the world with a total global production of 124.246 million tons in 2016.^1^ In 2019, fruit production was 157 million tons worldwide.^2^ Apart from the fresh fruit and its juice, pectin and essential oils are also important commercialized products of citrus (Fisher and Phillips, 2008). Commercially, several species fall under the term citrus, including lemons, limes, mandarins, satsumas, clementines, common mandarins and tangerines, oranges, grapefruits, and pummelos (Zhong and Nicolosi, 2020). The non-existence of genetic diversity in many commercially cultivated crops (because of monoculture) has made them more susceptible to biotic and abiotic stresses (Esquinas-Alcázar, 2005; Keneni et al., 2012). Citrus trees are susceptible to many pathogens including nematodes, fungi, oomycetes, bacteria, spiroplasmas, phytoplasmas, viruses, and viroids, and the main abiotic stresses affecting these trees are acid, alkaline, and salty soils, flooding and drought, freezing, and high temperatures.

Citrus trees have complex reproductive biology. The apomixis present in citrus, which means that adventitious embryos initiate directly from maternal nucellar cells, limits the development of less vigorous zygotic embryos. They also have long juvenile periods and require at least 5 years for the start of the flowering phase in subtropical areas, and usually, several years more to achieve fully mature characteristics. The complex taxonomic relationships among cultivar groups are another difficulty and one of the reasons for the low-level impact of conventional breeding in citrus genetic improvement (Gmitter and Talon, 2008). Genetic transformation offers an excellent strategy for the genetic enhancement of citrus since it is based on the introduction of specific traits into known genotypes without altering their elite genetic background. Biotechnological tools have assisted in the fast germplasm improvement of current cultivars (Peña and Navarro, 2000) and the development of new varieties.

This review provides insights into the most relevant aspects of genetic transformation of citrus species including explant selection, biological and physical methods for transformation, and dependence on the genotype. We explore the possibilities for the promoter, selection, and reporter systems, and discuss novel and emerging technologies aimed to get more acceptable biotechnological products with no integration of exogenous DNA (“DNA-free”), using cisgenesis, intragenesis, and gene-editing.

## Overview

The genus *Citrus* belongs to the subfamily Aurantoidea. Historically, within this subfamily there have been three genera of economic importance, namely *Fortunella*, *Poncirus*, and *Citrus*; however, more recently, it has been suggested they all belong to *Citrus* (Mabberley, 2004). Considering an evolutionary perspective, the four taxa identified as the ancestors of most of the cultivated citrus are *Citrus medica* L. (citron), *Citrus reticulata* Blanco (mandarin), *Citrus maxima* (Burm.) Merr. (pummelo), and *Citrus micrantha* Wester (papeda) (Wu et al., 2018; Ahmed et al., 2019). The secondary species, which result from successive natural hybridizations between the four fundamental species, are *Citrus sinensis* (L.) Osb. (sweet orange), *Citrus aurantium* L. (sour orange), *Citrus paradisi* Macf. (grapefruit), *Citrus limon* (L.) Burm. (lemon), *Citrus jambhiri* Lush (rough lemon), and *Citrus aurantifolia* (Christm.) Swing. (lime) (Wu et al., 2018; Ahmed et al., 2019). Finally, modern commercial cultivars come from artificial hybridizations: the rootstocks Carrizo and Troyer citrange hybrids (sweet orange × *Poncirus trifoliata*) and Swingle citrumelo (grapefruit × *P. trifoliata*) (Peña et al., 2008).

Although *Poncirus* and *Citrus* genera are the most amenable for *in vitro* regeneration (Vardi et al., 1982; Kobayashi et al., 1983), all species, hybrids, and economically important cultivars show a high degree of genotype-dependent variability in the efficiency of genetic transformation and regeneration (Bond and Roose, 1998).

## *Agrobacterium*-Based Transformation Methods on Citrus

Citrus crops are not naturally susceptible to *Agrobacterium tumefaciens*. Although citrus are generally recalcitrant to *Agrobacterium*-mediated transformation, researchers have successfully achieved the recovery of transgenic plants for many genotypes (Peña et al., 2008). In general, the transformation efficiencies achieved using *Agrobacterium* can range from 0 to 45% for most citrus cultivars (Febres et al., 2011). The most common disarmed *Agrobacterium* strains used for the transformation of citrus species and relatives are the octopine strain LBA4404 (Kaneyoshi et al., 1994; Ali et al., 2012) the nopaline strain C58 (Bond and Roose, 1998), and the agropine strains EHA101 or EHA105 (Moore et al., 1992; Peña and Navarro, 2020). *A. tumefaciens* A281 (the oncogenic ancestor of EHA105) produced the earliest and the highest frequency of tumor formation either in epicotyls or stem segments of Pineapple sweet orange, Mexican lime, Clemenules clementine, Carrizo citrange, *P. trifoliata*, Fino lemon, Cleopatra mandarin, *Citrus macrophylla*, sour orange and Mediterranean mandarin (Cervera et al., 1998a,^2005^; Fagoaga et al., 2005; Peña et al., 2008). Successful transformation of embryogenic calli from Ponkan mandarin and Valencia sweet orange has been also attainable using strain EHA105 (Li et al., 2002; Peña et al., 2008). Transformation efficiency from different strains is mainly attributable to Ti plasmids and specifically to the *vir* region present on them. The study of Ghorbel et al. (2001) have added extra copies of *virG* genes from pTiBo542 (Ti plasmid contained in EHA105) to strain C58, which resulted in a significant increment in transformation frequencies of C58 in several citrus genotypes.

Optimizing *A. tumefaciens*-explant co-cultivation conditions is always essential to enhance citrus transformation efficiency. The main parameters to adjust are bacterial inoculation and co-cultivation time, bacterial concentration, medium composition, and light-darkness conditions (Febres et al., 2011). The *Agrobacterium* inoculation times range between 5 min (Molinari et al., 2004) and 20 min (Yang et al., 2000; Almeida et al., 2003a). However, incubation periods greater than 10 min have led to an increased number of shoot escapes and a reduction in transformation efficiency (Costa et al., 2002). The bacterial inoculum concentration varies between 4 × 10^7^ (Peña et al., 1995b; Yu et al., 2002) and 5 × 10^8^ cfu/ml (Kaneyoshi et al., 1994; Bond and Roose, 1998; Luth and Moore, 1999) depending on the citrus cultivar. A very low amount of bacteria reduces transformation efficiency but an excess stresses the plant cells (Costa et al., 2002; Yu et al., 2002). The co-cultivation time is usually 2 or 3 days, with an increase in the transformation efficiency with longer co-cultivation periods (Cervera et al., 1998b). Co-cultivation periods of more than 5 days, however, often lead to an overgrowth of *Agrobacterium*, which decreases the regeneration efficiency. Finally, optimal co-cultivation temperature varies between 19°C (Li et al., 2002, 2003) and 28°C (Luth and Moore, 1999).

Callus cells derived from cambium tissues are the most competent for regeneration (Peña et al., 2004a). Treatments favoring the development of such callus tissue as co-cultivation in a culture medium rich in auxins and incubation of the explants in darkness for the first 2–4 weeks after bacterial inoculation, greatly increased transformation frequencies (Cervera et al., 1998b). The auxin 2,4 dichlorophenoxyacetic acid (2,4-D) in co-cultivation medium led to the highest effects allowing de-differentiation of citrus cells and taking them to a competent state for stable transformation in many cultivars including sour orange, sweet orange, lime, and Troyer citrange (Ghorbel et al., 2000; Peña et al., 2004a,b; Rai, 2006).

Some reports have discussed the need for a pre-culturing step (Spencer and Towers, 1991; Costa et al., 2002). This step, however, has been replaced for the addition of acetosyringone to the bacterial inoculum and the co-cultivation medium, thus promoting transcription of *A. tumefaciens* virulence genes (Kaneyoshi et al., 1994; Cervera et al., 1998a).

## Physical-Based Transformation Methods on Citrus

### Biolistic for the Transformation of Citrus Epicotyl Explants

Different methods have been adapted from the original publication by Sanford et al. (1987) to the biolistic-mediated transformation of plant cells. For citrus species, the study of Yao et al. (1996) have reported transformation of tangelo using non-differentiated embryonic callus and therefore demonstrated the integration of transgenes in this species. The study of Bespalhok Filho et al. (2003) performed epicotyl bombardment with GUS as the reporter gene, observing expression in meristematic cambial tissue of Carrizo citrange. In all cases, plant regeneration represented a challenge, since citrus explants are recalcitrant to produce roots, and consequently, to obtain a plant comparable to those established in greenhouses or orchards.

**FIGURE 1:**
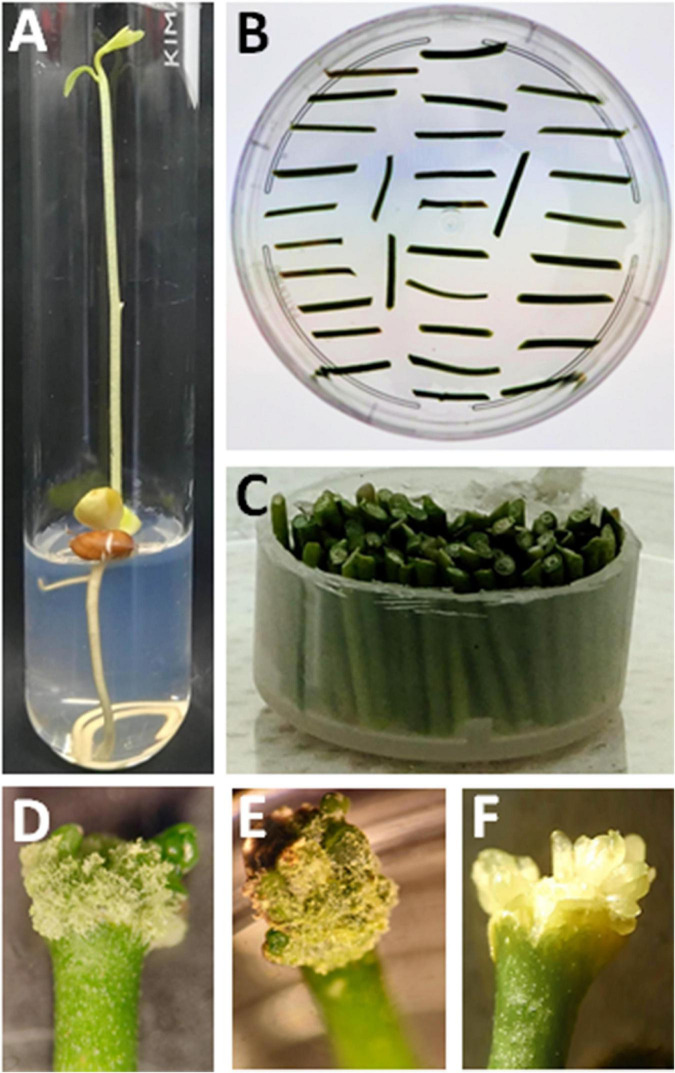
Bombardment and regeneration of Citrus explants. **(A)** Germinated seedlings *in vitro*; **(B)** Cut of epicotyls. **(C)** Arrangement of epicotyls in a plastic ring for bombardment. **(D,E)** Callus in apical regions of the explants produced in the dark after transformation. **(F)** Photosynthetic somatic embryos with apical dominance were produced after light exposure.

The work of Wu et al. (2016) has described the transformation of epicotyls in Carrizo citrange. In this method, epicotyl explants were bombarded at their apical region with microprojectiles coated with the DNA of interest. The DNA enters the cells, where it is expected to enter the nucleus and can be stably inserted into the genome. Callus formation was developed *via* tissue culture to obtain regenerated plantlets, which are finally grafted to generate a complete plant. The steps of the biolistic transformation protocol are illustrated in Figure 1.

Elongated epicotyl stems are adapted to photoperiod and then used as explants for bombardment with gold or tungsten particles coated with DNA. The decision of using linear or circular DNA (plasmid) will depend on the strategy. For example, if the intention is to induce homologous recombination, the DNA should be linearized (Tomas et al., 1995; Cabrera-Ponce et al., 1997). Otherwise, a circular DNA, either in its supercoiled or relaxed form, can be efficiently transformed. The bombardment of microparticles for citrus tissue has been assayed with pressure from 900 to 2,000 psi but in general, citrus stems bear resistance to the accelerated particle impact. However, the transformation of apical stem tissues improves transformation efficiency. After bombardment, visible photosynthetic tissue emerges from the stem ends as well as from the middle portion of the stem, although in a lower proportion. In a period of 2 months, the embryos can be dissected for further heterografting. The regeneration of plants with a radicular system is a technical challenge. For this reason, grafting of the plantlet onto a vigorous rootstock is advisable. Enhanced transformation efficiency and reduction of escapes were achieved by Wu et al. (2019), with a biolistic strategy based on phosphomannose isomerase (PMI) and mannose selection (see section “Selection Markers, New Reporter Genes, and Marker-Free Systems for Citrus Transformation” for selection markers).

### Magnetofection of Pollen With DNA-Functionalized Nanoparticles

Pollen magnetofection is a new technique that has been used to produce transgenic seeds without *in vitro* plant regeneration. This technique was successfully applied to cotton and tomato pollen-based transformation (Zhao et al., 2017; Zhang et al., 2019; Marcelino-Perez et al., 2021) and consists in the generation of nanoparticles and their functionalization to DNA or RNA encoding for the trait of interest, to generate magnetic nanoparticles (MNPs) which are then introduced into pollen through the application of a magnetic field. This goal is technically efficient since MNP-DNA/RNA complexes interact *via* electrostatic attractions. Specifically, 1 to 10 million pollen particles can be mixed with the loaded MNPs and placed on a magnetic plate, where they can enter into the pollen *via* its natural pores. Pollen must be used immediately to pollinate emasculated flowers. It should be considered that pollen viability is measured in hours; therefore, the treated pollen should be used immediately. Seeds obtained through pollination with magnetofected pollen can enclose the transgene of interest. Indeed, exogenous DNA is integrated into the plant genome and is inherited in a Mendelian manner.

Pollen magnetofection could be applied to citrus, thus facilitating the generation of genetically modified plants. Moreover, this technique is potentially useful for gene editing as well. Despite the accessibility in introducing this technique for citrus transformation, a series of considerations must be taken (Xoconostle-Cázares, personal communication). Plants should come from certified orchards and should be maintained in a biosafety greenhouse with full irrigation, fertilization, and a light/dark regime of 18:6 h. Flowering can be naturally or artificially induced and production from 20 to 100 flowers can be achieved in a period of 3 months. *C. aurantifolia* (Mexican lime) grown in tropical orchards, can produce flowers seven to eight times in a year, while *C. sinensis* (sweet orange) can flower up to three times in the same period. In a regular magnetofection experiment, 100 flowers can be pollinated. After approximately 3 months, mature fruits would be ready for harvesting. The surface of mature fruits should be smooth. The harvesting of mature fruits will allow the recovery of mature transformed seeds, which can be lately germinated in a seedling nursery at 30°C. Plantlets emerging from the nursery can be then transferred to individual pots for further analyses. An average of five positive plantlets carrying out the transgene in a regular experiment of one hundred pollinated flowers is expected. The transformation efficiency could be influenced by fruit quality. Transgenic plants should be maintained under biosafety conditions.

### Polyethylene Glycol-Based Transformation and Electroporation

An alternative strategy for incorporating DNA into plant citrus cells is the transformation of protoplasts. The use of protoplasts, either for cell fusion or for DNA uptake, is a widely employed technology with great potential in the field of citrus genetic improvement (Grosser and Gmitter, 1990, 2011). One of the methods for protoplast transformation is stimulation with polyethylene glycol (PEG), a treatment that induces the agglutination of protoplasts and subsequent incorporation of DNA (or other macromolecules) *via* endocytosis (Kobayashi and Uchimiya, 1989; Vardi et al., 1990).

In the pioneering early protocols, antibiotic resistance genes were the selection markers of choice. Later, PEG-mediated citrus transformation included the expression of green fluorescent protein (GFP) in order to identify the transformed calli to be subsequently cultured in somatic embryogenesis induction media (Fleming et al., 2000; Olivares-Fuster et al., 2003; Guo and Grosser, 2004; Omar et al., 2007). PEG-mediated transformation is also particularly useful for citrus varieties that offer difficulties for genetic transformation *via Agrobacterium*, like mandarin hybrids (Dutt et al., 2018a; Omar et al., 2018). This strategy has been recently employed for inducing biallelic and homozygous mutations *via* genome editing of embryonic protoplast cells (Huang et al., 2020). DNA can also be introduced into protoplasts by electroporation through destabilization of plasma membranes and the formation of pores. This strategy has been employed for citrus genetic transformation of Ponkan mandarin (Hidaka and Omura, 1993) and Hamlin sweet orange (Niedz et al., 2003).

## *In vitro* Regenerations of Transgenic Citrus Plants

Regeneration competence is the first limitation for the production of transgenic plants and many recalcitrant species actually have very low or null regeneration frequencies (Peña et al., 2004a). García-Luis et al. (1999) have proven that citrus genotypes, culture conditions, and medium composition determine the regeneration pathway (García-Luis et al., 1999; Bordón et al., 2000; Moreira-Dias et al., 2000).

Regeneration of whole transgenic citrus plants has been achieved through either organogenesis (direct or indirect) or somatic embryogenesis (Chiancone and Germanà, 2012). *In vitro* plant organogenesis from epicotyl and internodal stem segments is the chosen strategy which has been applied to several citrus genotypes, including Carrizo citrange, Troyer citrange, sweet orange, Mexican lime, grapefruit, Swingle citrumelo, and *P. trifoliata* (Peña et al., 2008). The effects of different factors, such as explant orientation, polarity, and cut surface contact with the medium as well as growth regulators treatments, were assessed in different studies in order to improve *in vitro* regeneration efficiency (Maggon and Deo Singh, 1995; Ghorbel et al., 1998; Pérez-Molphe-Balch and Ochoa-Alejo, 1998; Germanà et al., 2008). Somatic embryogenesis has been used for regeneration of a few transformed species including *C. sinensis* and *C. reticulata* (Li et al., 2002; Peña et al., 2008) by using different types of explants such as protoplasts and embryogenic calli (Niedz et al., 1995; Li et al., 2003). The interest in somatic embryogenesis is based on the high regeneration efficiency obtained and in the rare occurrence of somaclonal variation (Henry, 1998).

The culture media used for regeneration by either organogenesis or embryogenesis should contain a series of components to facilitate the formation of calli or buds from transformed explants, but with the minimum of escapes. For that reason, regeneration media normally contain an antibiotic agent for selection (see section “Selection Markers, New Reporter Genes, and Marker-Free Systems for Citrus Transformation” for marker selection), since only a few cells put in contact with the transformation vector are effectively transformed. Peña et al. (1995a,b, 1997) proposed the cultivation of explants in darkness for 2–4 weeks in regeneration/selection medium after co-cultivation with *A. tumefaciens* for the generation of a higher number of transformed buds (Domínguez et al., 2004; Peña et al., 2004b). Moreover, Moreira-Dias et al. (2000) showed that the addition of the cytokinin 6-benzylaminopurine (BAP) was a requisite for optimal shoot regeneration from Troyer citrange explants, while the influence of auxins appeared to be non-significant. The authors reported no callus and very few bud formations at the apical end of the explants in the absence of BAP and that most of the calli remained quiescent without becoming a shoot. Peña et al. (2004b) also demonstrated that co-cultivation in a BAP-supplemented media promoted a faster differentiation response and multiple bud formation in experiments of indirect organogenesis of Carrizo citrange.

**FIGURE 2:**
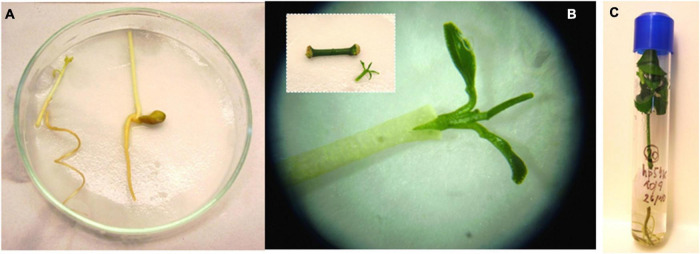
Shoot tip grafting procedure. **(A)** Decapitated etiolated epicotyls to be used as rootstocks. **(B)** Micrografting of a transformed shoot using a stereoscopic microscope. INSET: shoot detached from the internodal segment. **(C)** *In vitro* growing of the grafted plantlet.

**FIGURE 3:**
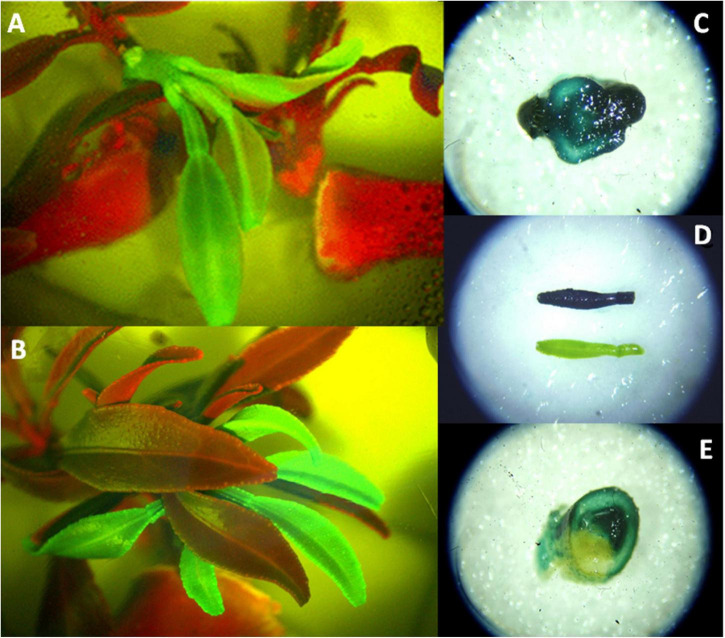
Reporter expression of transformed citrus explants. **(A,B)** GFP expression of transgenic *C. sinensis* shoots (green). Red shoot and internodal segments are non-transgenic tissue. **(C)** GUS expression in a disk section from transgenic shoot base. **(D)** GUS positive (blue) and negative (green) leaves coming from transformed explants. **(E)** GUS expression in a disk section from a chimera shoot.

The rooting of shoots and embryos is the most challenging step of citrus transgenic production, strikingly reducing transformation efficiencies. *P. trifoliata*, grapefruit, and Swingle citrumelo have been successfully rooted in naphthalene-acetic acid (NAA) supplemented media (Kaneyoshi et al., 1994; Luth and Moore, 1999; Molinari et al., 2004). *C. macrophylla*, Cleopatra mandarin, sour orange, and Mexican lime have been also efficiently rooted in medium supplemented with indole-3-butyric acid (IBA) (Tallón et al., 2012). The use of micrografting or shoot tip grafting to recover plants from transformed shoots or buds, however, has become a routine procedure, with important increases in transformation efficiencies (Peña et al., 1995a,b). This technique involves placement of the shoot tip explant onto a decapitated rootstock, which is generally an etiolated Troyer citrange epicotyl (Figure 2; Navarro et al., 1975; Peña and Navarro, 2000). The regenerated shoot apical end has to be in contact with the vascular ring of the rootstock. Grafted shoots should have previously tested positive for reporter marker activity (GFP or β-D-glucuronidase, GUS, Figure 3), denoting the transgenic nature of the explant. Micrografted shoots or buds are then cultured in a liquid nutrient medium for proper growing. Subsequently, a new grafting of the *in vitro*-grown plantlets on vigorous rootstocks in the greenhouse allows rapid acclimatization (Peña et al., 2008).

The micrografting methodology has been efficiently used for whole plant generation from shoots derived from organogenesis as well as from germinated somatic embryos (Niedz et al., 2003; Olivares-Fuster et al., 2003). Some of the different citrus genotypes in which this technique was applied include *C. sinensis*, *P. trifoliata*, *C. aurantifolia*, *C. aurantium*, and grapefruit (Kobayashi et al., 1996; Bond and Roose, 1998; la Malfa et al., 2000; Wong et al., 2001; Januzzi Mendes et al., 2002; Yu et al., 2002; Almeida et al., 2003a,b; Boscariol et al., 2003; Iwanami et al., 2004; Endo et al., 2005; Fagoaga et al., 2005).

## Genotype Dependence of Citrus Transformation

The efficiency of genetic transformation and regeneration highly depends on the genotype of all cultivated natural or hybrid species of the genus *Citrus* and its relatives (Bond and Roose, 1998). As well as for *P. trifoliata* (Kaneyoshi et al., 1994), the studies by Peña et al. (1995a), Cervera et al. (1998b), and Yu et al. (2002) have developed highly efficient methods for the transformation of modern commercial hybrid cultivars, such as Carrizo and Troyer citrange rootstocks using *A. tumefaciens* followed by *in vitro* regeneration and shoot tip grafting.

Other pieces of research have reported biolistic transformations (Wu et al., 2016) and transformation of mature tissues of several varieties including hybrids such as US-942 (*C. reticulata* × *P. trifoliata*) and Flying Dragon (Marutani-Hert et al., 2012). Other commercial hybrids currently used, such as swingle citrumelo and tangelo (*C. reticulata* × *C. paradise*), are also susceptible to *Agrobacterium*-mediated transformation (Molinari et al., 2004; De Oliveira et al., 2009).

Among the natural hybrids or secondary species, sweet orange has been extensively studied, and numerous protocols were published mainly based on the *Agrobacterium*-mediated methodology (Peña et al., 1995c; Bond and Roose, 1998; Yu et al., 2002; Li et al., 2003; Cervera et al., 2005; De Oliveira et al., 2009; Fávero et al., 2012), protoplast transformation (Fleming et al., 2000) and embryogenic suspension cells (Dutt and Grosser, 2010). For sour orange, Gutiérrez-E et al. (1997), as well as Ghorbel et al. (2000), developed an efficient transformation strategy, also based on *Agrobacterium* co-cultivation. Another natural hybrid used as rootstock is rough lemon, which was genetically transformed by Savita et al. (2011) and Ali et al. (2012). Other crops, such as Mexican lime (Peña et al., 1997; Pérez-Molphe-Balch and Ochoa-Alejo, 1998; Domínguez et al., 2000; De Oliveira et al., 2015), Femminello siracusano lemon (*C. limon* (L.) Burm. F) (Gentile et al., 2007) and grapefruit (Yang et al., 2000; Costa et al., 2002; Jia et al., 2017) have been efficiently transformed and regenerated.

*Agrobacterium*-mediated conventional transformation methods, using either juvenile or mature tissue explants showed low efficiency in mandarins and clementines (Cervera et al., 1998b). However, some more recent pieces of research have reported optimized methods (Li et al., 2002; Khawale et al., 2006; Cervera et al., 2008) and alternative strategies based on the use of cell suspensions and protoplast transformation (Dutt et al., 2018a).

To date, several biotechnological developments have been reported for different citrus species and varieties (see detailed information in Table 1).

**TABLE 1:** Main biotechnological developments in citrus or relative species.

Citrus Species/Variety	Trait	Gene of interest	Greenhouse/Field trial	Strategy	References
*C. sinensis*	Resistance to Citrus Canker	CsLOB1	G	Gene editing	Peng et al., 2017
*C. sinensis*		WRKY22	G	Gene editing	Wang et al., 2019
*C. sinensis*		hrpN	G	Overexpression	Barbosa-Mendes et al., 2009
*C. paradisi*		CsLOB1	G	Gene editing	Jia et al., 2016, 2017
*C. sinensis*		Attacin A	G	Overexpression	Boscariol et al., 2006; Cardoso et al., 2010
*C. sinensis*		MdSPDS1	G	Overexpression	Fu et al., 2011a
*C. sinensis*		Dermaseptin	G	Overexpression	Furman et al., 2013
Troyer citrange		Snakin-1	G	Overexpression	Conti et al., 2020
*C. sinensis*		Peroxidase25	G	Overexpression	Li et al., 2020
*C. sinensis* and Carrizo citrange		FLS2 receptor	G	Overexpression	Hao et al., 2016a
*C. sinensis*		Bs2	G	Overexpression	Sendín et al., 2017
W. Murcott mandarin		Xa21	G	Overexpression	Omar et al., 2018
Carrizo citrange	Resistance to Citrus Canker and Huanglongbing	M-thionin	G	Overexpression	Hao et al., 2016b
*C. sinensis*	Resistance to Huanglongbing	NPR1	F	Overexpression	Dutt et al., 2015
*C. sinensis*		Cecropin B	G	Overexpression	Zou et al., 2017
*C. aurantifolia*		β-defensin 2 and Lysozyme	G and F	Overexpression	Guerra-Lupián et al., 2018
*C. sinensis*		SAMT1	G	Overexpression	Zou et al., 2021
*Citrus sp.*		SOD2 and SOD7	G and F	Overexpression	EPA-HQ-OPP-2014-0834, F.-9926-99, 2015
*C. sinensis*	Reduced attraction to *Diaphorina citri*	(E)-β-caryophyllene synthase	G	Overexpression	Alquézar et al., 2021
*C. sinensis*	Resistance to CPsV	CPsV coat protein	G	RNA silencing	Reyes et al., 2011
*C. aurantifolia*	Resistance to CTV	CTV p25 coat protein	F	Overexpression	Domínguez et al., 2002
*C. aurantifolia*		CTV p23 from CTV	F	Overexpression	Soler et al., 2012
*C. aurantifolia*	Drought and Salinity Tolerance	CBF3	G	Overexpression	Romero-Romero et al., 2020
*C. sinensis*	β-carotene content synthesis	Csβ-CHX	G	RNA silencing	Pons et al., 2014

## Types of Citrus Explants Used for Genetic Transformation

A variety of explants, such as internodal stem segments, epicotyls, cotyledons, leaf segments, protoplasts, and embryogenic calli from different citrus species and relatives, have been assessed for *Agrobacterium*-mediated transformation (Moore et al., 1992; Almeida et al., 2003a; Kayim et al., 2004; Khawale et al., 2006; Omar et al., 2007; Chen et al., 2008; De Oliveira et al., 2009, 2015; Ballester et al., 2010; Duan et al., 2010; Fu et al., 2011a,b; Ali et al., 2012). All of them have their intrinsic advantages and drawbacks.

The first report of citrus transformed material was of callus tissue formed from primary explants (Grinblat, 1972; Chatuverdi and Mitra, 1974; Barlass and Skene, 1982; Edriss and Burger, 1984). The addition of growth regulators, mainly the cytokinin BAP, has shown a good response for this kind of explants. However, embryogenic calli lose their regeneration capacity when they are sub-cultured for long periods.

Juvenile internodal stems explants are one of the most prevalent and efficient starting materials for citrus transformation (Orbović and Grosser, 2015). The availability of this kind of explants and the higher regeneration and transformation frequencies are the major advantages over the use of mature tissue explants. The generation of plants using this material, however, would take more than 10 years to flower and fruit, which drastically prolongs the time required to analyze newly introduced traits. The optimization of transformation techniques that bypass the juvenile stage could reduce the time and costs involved in evaluating transgenic new traits (Almeida et al., 2003a; Cervera et al., 2005, 2008). Mature citrus tissue explants help to reduce flowering time but suffer from a considerable decline in regeneration frequency (50–70% less) and transformation potential, therefore it is necessary to carefully select material and adjust tissue culture conditions and media composition (Von Aderkas and Bonga, 2000). As of today, mature materials from sweet orange (Cervera et al., 1998a,^2005^), sour orange (Ghorbel et al., 2000), lime, and some mandarin genotypes have been successfully transformed and regenerated. A selection of stem pieces from the first flushes of propagated adult buds was the choice for transforming mature tissue. In this regard, transgenic sweet orange plants regenerated from mature tissues flowered and produced fruits 14 months after transferring to the greenhouse (Cervera et al., 2005).

The study of Kaneyoshi et al. (1994) have established the first efficient protocol for the transformation of *in vitro* seedling material and applied it to the generation of transgenic *P. trifoliata* plants. By using 1 cm long etiolated epicotyl segments from 20-day-old grown *in vitro* as starting material for transformation, they demonstrated that these kinds of explants were highly responsive to shoot regeneration, with the extra advantage of not requiring explant disinfection steps. Peña et al. (1995b) used a similar protocol to transform Carrizo citrange and Gutiérrez-E et al. (1997) to transform bitter orange (*C. aurantium*) and Key lime (*C. aurantifolia*). Similar protocols were applied to transform epicotyl segments from *C. sinensis* commercially important cultivars including Washington navel, Valencia, Hamlin, Pera, and Natal (Bond and Roose, 1998; Luth and Moore, 1999; Boscariol et al., 2003), Original protocol from Kaneyoshi et al. (1994) suffered different modifications including longitudinal cuttings of the epicotyl segments (in two halves) to enhance regeneration and transformation frequency (Yu et al., 2002) or cutting of transversally thin layers of about 1–2 mm (Le et al., 1999). All the changed conditions only reduced transformation efficiency compared with the use of 1 cm long explants probably due to *Agrobacterium* overgrowth and toxicity of such small explants (Molinari et al., 2004). Lately, Costa et al. (2002) and Febres et al. (2003), efficiently transformed *C. paradisi* cv. Duncan and De Oliveira et al. (2015) also established a similar protocol using 30-day-old epicotyls for the transformation of Mexican lime.

Leaf explants have been also tested for genetic transformation either for direct organogenesis or going through an intermediate process of callus formation (Moore et al., 1992; Khan et al., 2009; Ali et al., 2012). Abundant and rapid accessibility to leaf disks from germinating seedlings and a lower risk of contamination make them a considerable option as starting material for *Agrobacterium*-mediated transformation. Mature leaf transformation allows introducing new traits without losing the clonal fidelity compared with epicotyls (Sandal et al., 2007). Moore et al. (1992) used citrus leaf disks as explants and compared their organogenic potential with stem segments. They reported that shoot production was much more effective when stems were used as explants compared with leaf segments, probably because organogenesis occurs with higher efficiency from stems (Almeida et al., 2003a).

Sweet orange was the first woody crop in which plant protoplasts were used for regeneration processes (Vardi et al., 1982; Kobayashi et al., 1983). Nowadays, protoplasts are used as starting material for most citrus species and relatives and were applied to produce somatic hybrid plants from more than 150 parental combinations (Grosser and Gmitter, 1990), thus contributing to germplasm expansion and improvement (Grosser et al., 1996, 1998a,b). It is well-known, that protoplast generation is a time-consuming and labor-intensive methodology but the use of this kind of culture as explants has a series of advantages. Protoplast transformation can circumvent the use of antibiotic-resistance genes and antibiotic selection, thus eliminating some public perception problems (Fleming et al., 2000). This system could subsequently be extended to other polyembryonic citrus cultivars, including seedless sweet oranges, lemons, or satsuma mandarins.

Among the citrus species, juvenile tissues from mandarin hybrids, including epicotyls, are the most difficult to infect and transform with *A. tumefaciens* (Cervera et al., 1998b), which results in low genetic transformation efficiency (Dutt et al., 2010). Direct incorporation of DNA into protoplasts using electroporation (Niedz et al., 2003) or PEG-mediated DNA uptake (Fleming et al., 2000; Omar et al., 2007) is an alternative to bypass *Agrobacterium*-mediated transformation problems in those genotypes. A clear example is the PEG-mediated transformation of W. Murcott tangor using protoplasts, which allows a considerable increment in the transformation efficiency compared with the conventional epicotyl-mediated *Agrobacterium* process (Dutt et al., 2018a). The use of protoplasts has been recently taken into consideration again, regarding their amenability for gene editing, with or without the use of DNA molecules, which minimizes the possibility of foreign DNA integration (Huang et al., 2020).

## Selection Markers, New Reporter Genes, and Marker-Free Systems for Citrus Transformation

Most of the *in vitro* citrus regeneration and transformation protocols need selectable marker genes (antibiotic or herbicide resistance) such as the *nptII* (Neomycin Phosphotransferase II) gene, in combination with kanamycin as a selective agent (reviewed in Peña et al., 2004a). Concomitant to the use of *nptII*, the product of expression of the *uidA* gene (GUS), has been widely applied as a co-expressed reporter gene to facilitate the selection of positive transformants (Moore et al., 1992; Peña et al., 1997; Cervera et al., 1998c; Domínguez et al., 2000). GUS has been extensively employed as a reporter gene for citrus genetic transformation (Moore et al., 1992; Peña et al., 1995b,^1997^; Gutiérrez-E et al., 1997). Its adequate use is important to avoid escapes and the incidence of chimeras (Gutiérrez-E et al., 1997; Yu et al., 2002; Figure 3E). However, Domínguez et al. (2002) have demonstrated that the detection of a high frequency of transformants was possible without *npt*II/*uidA* selection. Indeed, once the regeneration of the positive transformants, which are detected by PCR-mediated analysis of regenerated roots, is achieved, antibiotic resistance is no longer necessary. But, in the case of *nptII* and *uidA* genes, other than remaining stably integrated into the genome for long term, they do not produce negative effects on crop characteristics (Pons et al., 2012).

To find other selectable marker genes suitable for citrus genetic transformation, Costa et al. (2002) have developed a protocol for grapefruit transformation based on the use of hygromycin as a selective agent. The *hpt* gene codes for a hygromycin phosphotransferase that, like the protein product of *npt*II, detoxifies aminoglycoside antibiotics by phosphorylation. Hygromycin selection, however, faces difficulties regarding the screening of transgenic tissues, because of the generation of escapes and chimeras (Padilla and Burgos, 2010). Resistance to phosphinotricine (Basta, Bialaphos, or glufosinate) for transgenic callus selection has been employed in ponkan embryogenic calli overexpressing *bar* gene (Li et al., 2002). Later on, Zhang Y. Y. et al. (2017) developed a transformation approach for pummelo (*C. maxima*) based on *in planta A. tumefaciens* infection and subsequent selective culture using hygromycin, Basta or kanamycin resistance. After PCR-based screening of regenerated shoots, efficiencies achieved were 20.41, 19.37, and 3.21% respectively. Recently, the study of Merritt et al. (2021) reported glyphosate-resistant Duncan grapefruit plants, obtained by inducing native EPSPS mutations.

The use of selectable marker genes for resistance to antibiotics and or herbicides has been a matter of public constraint (Miki and McHugh, 2004) and in some cases has been shown to reduce regeneration capacity (Moore et al., 1992). Therefore, researchers developed alternative methods for screening and selecting transformed tissues. In this regard, GFP and its enhanced derivatives (EGFP) have been extensively employed as reporter genes for the selection of transgenic plant tissues (Chiu et al., 1996; Stewart, 2001) in *Agrobacterium-mediated* transformation (Ghorbel et al., 1999; Fleming et al., 2000; Yu et al., 2002; Cervera et al., 2008), in protoplast transformation (Guo and Grosser, 2004; Omar and Grosser, 2008), for biolistic assays (Wu et al., 2016) and gene editing (Huang et al., 2020). A novel reporter system based on the measurement of increased anthocyanin accumulation by overexpression of *Ruby* and VvMYBA1 transcription factors has been used to detect *Agrobacterium*-mediated Mexican-lime transformed explants (Dutt et al., 2016) or by overexpressing VvMYBA1 in protoplasts, under the control of embryo-specific Dc3 promoter (Dutt et al., 2018b).

Numerous alternative methods have been developed in order to replace the selection systems based on antibiotics resistance. The PMI system (PMI/mannose) is a positive selection strategy based on the expression of the *man*A gene from *Escherichia coli*, which is able to metabolize mannose to fructose-6-phosphate. When the only carbon source for explants in the selective culture is mannose, the positive transgenic tissues will be capable of growing, whereas non-transformed tissues will have growth arrest due to carbon starvation (Wang et al., 2000). Boscariol et al. (2003) have used this system for the selection of transformed sweet orange tissues coming from *Agrobacterium*-mediated transformation of epicotyl segments and achieved transformation efficiencies of 3–23% depending on the variety used. Subsequently, Ballester et al. (2008) achieved higher or similar transformation rates for citrange (30%) and sweet orange (13%) using *in vitro*-germinated seedlings as a source of epicotyl segments. The PMI/mannose positive selection system combined with EGFP based monitoring of transformed tissues allowed early elimination of escapes in Carrizo explants (Dutt et al., 2010). In biolistic assays, this alternative system has been successfully applied in Carrizo citrange, with efficiencies of 1.9% positive shoots per shot (Wu et al., 2019).

Concomitantly to the use of alternative selection methods that do not rely on antibiotic resistance genes, the possibility of removing the marker gene once the transgenic plants have been recovered is suitable for woody species, where seedless propagation is usually observed, and long juvenility increase time periods required for getting segregation results on transgenic progenies. The multi-auto-transformation (MAT) vector system enables the production of marker-free transgenic plants by a combination of a positive selection mediated by the isopentenyl transferase (*ipt*) gene and a site-specific DNA recombination tool (Sugita et al., 1999). The enzyme *ipt* is involved in the production of cytokinins that induce cell division and the overproduction of transgenic shoots. The second component of the MAT system is a site-specific recombinase (R/RS) that removes DNA sequences located between RS recognition sites after transformation. The RS sites flank the *ipt* marker and the R recombinase transgenes, to facilitate the elimination of the selection marker system after cell transformation. In citrus, the system has been successfully applied in sweet orange, but not in citrange, where a high proportion of chimeras and erroneous sequence recombination occurred (Ballester et al., 2007). The addition of an indolacetamide-hydrolase and tryptophan-monooxygenase (*iaa*M/H) marker gene and an inducible promoter for controlling the site-specific recombinase R rendered higher efficiency rates (Ballester et al., 2008). Zou et al. (2013) reported the use of other marker-free transformation systems in citrus, based on the expression of Cre/*lox*P site-recombination coupled to the *ipt* selectable marker gene. The GFP reporter gene was inserted outside the *lox*P sequences with the aim of monitoring the rate of transformation and deletion efficiencies. The results obtained demonstrated that Cre/*lox*P-mediated excision was highly effective and accurate for Jincheng sweet orange. Marker-free transgenic Tarocco blood orange overexpressing antibacterial peptide gene AATCB, which conferred enhanced resistance to citrus canker, was obtained by using a Cre/*lox*P mediated recombination system combined with *ipt* positive selection. Transformation efficiency achieved was 21.4% (Peng et al., 2015). Recently, Peng et al. (2021a) described a similar strategy to confer citrus canker resistance but by co-transformation and sequential re-transformation of Tarocco blood orange with the same AATCB gene and another antimicrobial peptide, PR1aCB. They also used Cre/*lox*P-mediated site-specific recombination system and *ipt* selection to get marker-free plants and confirmed that double transformants showed enhanced citrus canker resistance.

## Promoter Sequences Used for Citrus Transgene Expression

The type of promoter used in the chimeric gene construct for plant transformation is essential to achieve adequate temporal or spatial regulated expression of the desired trait. Although the number of promoter sequences is rather limited, the selection of an adequate promoter is not a trivial issue (reviewed in Smirnova and Kochetov, 2020). Different promoters derived from virus, bacteria, or plant species have been employed for citrus genetic transformation. Table 2 displays a list of promoter sequences used in citrus, considering the species of origin and the regulated gene.

**TABLE 2:** Promoters used in citrus genetic transformation.

Type of expression	Promoter	Source	Transformed plant	Controlled gene	References
Constitutive	35S	CaMV (Cauliflower mosaic virus)	Sour orange	CTV coat protein	Gutiérrez-E et al., 1997
			Sour orange	CTV coat protein	Ghorbel et al., 2000
			Pineapple Sweet orange	GUS	Peña et al., 1995a
			Mexican lime		Peña et al., 1997
			Carrizo citrange		Cervera et al., 1998c
			Pineapple Sweet orange		Cervera et al., 1998a
			Washington Navel Sweet orange		Bond and Roose, 1998
			Citrange	AP1 and LFY	Peña et al., 2001
			Itaborai Sweet orange	GFP	Fleming et al., 2000
			Pineapple Sweet orange	CPsV hrps* for 54k, coat protein and 24k	Reyes et al., 2011
			Mexican lime	CTV hrps* for p20, p23 and p25	Soler et al., 2012
			Pineapple Sweet orange	Dermaseptin	Furman et al., 2013
			Troyer citrange	StSnakin-1	Conti et al., 2020
			Duncan grapefruit	PtFT1-scFv	Sinn et al., 2021
			Wanjincheng Sweet orange	CsSAMT1	Zou et al., 2021
	34S		Duncan grapefruit	CTV coat protein, RdRp and genomic 3′ end RNA	Febres et al., 2003
	MAS	*Agrobacterium tumefaciens*	Jincheng and Newhall Navel Sweet orange	Shiva A and Cecropin B	He et al., 2011
	Full length CsCYP, CsGAPC2, and CsEF1	Sweet orange	Hamlin Sweet orange	GUS	Erpen et al., 2018
	Partial CsCYP, CsGAPC2, and CsEF1		*Nicotiana benthamiana*		Corte et al., 2020
	YAO	*Arabidopsis thaliana*	Carrizo citrange	PDS	Zhang et al., 2017
Embryo-specific	Dc3	Carrot	Hamlin Sweet orange	VvMybA1	Dutt et al., 2018b
Fruit-specific	CitMT45	Satsuma mandarin	Valencia Sweet orange	GUS	Endo et al., 2007
Pulp and flower-specific	Cl111	Acid lemon	Acid lemon and acidless lime		Sorkina et al., 2011
Flower-specific	CitSEP, CitWAX, CitJuSac, CitVO1, and PamMybA	Sweet orange	Micro-tom tomato		Dasgupta et al., 2020
Seed-specific	CuMFT1	Satsuma mandarin	Trifoliate orange, satsuma mandarin, Kishu mikan and *Arabidopsis thaliana*		Nishikawa et al., 2008
Xylem vessels-specific	CsPP	Madam Vinous orange	Tobacco and Valencia orange		De Azevedo et al., 2006
Phloem-specific	rolC	*Agrobacterium rhizogenes*	Mexican lime	GUS	Dutt et al., 2012
	RTBV	*Rice Tungro Bacilliform Virus*			
	RSs1	*Oryza sativa*			
	AtSUC2	*Arabidopsis thaliana*			
	AtSUC2, AtPP2	*Arabidopsis thaliana*	Hamlin, Pera and Valencia Sweet orange		Miyata et al., 2012
	CsPP2	Sweet orange			
	AtSUC2, AtPP2	*Arabidopsis thaliana*	Hamlin and Valencia Sweet orange	Attacin A	Tavano et al., 2019
	CsPP2	Sweet orange	Carrizo citrange	CcFT3	Soares et al., 2020
	AtSUC2	*Arabidopsis thaliana*			
	GRP1.8	*Phaseolus vulgaris*	Tarocco blood Sweet orange	Cecropin B	Zou et al., 2017
	CsPP2.B1 and CsVTE2	Sweet orange	Carrizo citrange	GUS	Bezerra et al., 2021
Pathogen-inducible	gst1	Potato	Mexican lime	hrpN	Barbosa-Mendes et al., 2009
			Pineapple Sweet orange	Bs2	Sendín et al., 2017
			Jincheng Sweet orange	GUS	Zou et al., 2014
	PPP1, hsr203J	Tobacco			
	PR5	Sweet orange	Troyer citrange	CsMAPK1	De Oliveira et al., 2013
Heat shock-inducible	AtHSP70BP	*Arabidopsis thaliana*	Duncan grapefruit, Valencia Sweet orange, Key lime, Carrizo citrange, Sour orange, and Meiwa kumquat	GUS	Jia and Wang, 2014b
Stress-inducible	AtRD29A	*Arabidopsis thaliana*	Duncan grapefruit and Valencia Sweet orange	CsAP1 and CsLFY	Orbović et al., 2021

*hrps: Hairpins to induce RNA silencing.

Over the last years, a lot of work has been done to increase the availability of promoters and other regulatory sequences, including the development of synthetic promoters for citrus genetic transformation and its application to new breeding techniques (reviewed in Ali and Kim, 2019).

## Early Flowering Induction to Reduce the Juvenile Phase of Transgenic Citrus

Long juvenile phases (often more than 5 years) are a major constraint to the success of transformation methods based on juvenile tissue explants and of conventional breeding programs. The development and commercial release of new varieties by traditional breeding may require a complete process that can range from 25 to 30 years (Caruso et al., 2020). The discovery of novel genes implicated in citrus precocious flowering is relevant for the improvement of interesting traits, either by transgenesis or by conventional breeding. Some strategies to reduce the long juvenile periods in citrus trees rely on the basis of the knowledge related to flowering pathways in *Arabidopsis thaliana*.

The first successful approach in citrus plants that was conducted to reduce generation periods consisted of the constitutive overexpression of *Arabidopsis* LEAFY (LFY) or APETALA1 (AP1) genes in juvenile seedlings (Peña et al., 2001). Both genes are involved in the induction of flowering and their individual expression induced early flowering and fruiting in transgenic citrange rootstocks. AP1, however, was more efficient than LFY, because LFY also induced abnormalities in the vegetative growth. Flowering Locus T (FT) citrus orthologue, CiFT, was ectopically overexpressed in *P. trifoliata* (Endo et al., 2005) with subsequent shortening of the juvenile period, but again, the vegetative growth and plant architecture were aberrant. Velázquez et al. (2016) developed a viral vector-based tool to induce early flowering: the Citrus Leaf Blotch Virus (CLBV) carrying AtFT or CiFT genes. Within 4–6 months of vector inoculation in different genotypes, flowering was initiated with no other phenotypic abnormalities. Furthermore, Soares et al. (2020) reported a novel strategy to induce precocious flowering by overexpressing the *Citrus clementina* (CcFT3) orthologue under the control of AtSUC2 phloem-specific promoter in Carrizo citrange rootstocks. This strategy led to plants with normal morphology that flowered 16 months after transformation and, when juvenile scions were grafted, earlier flowering was also induced.

Other alternatives have been employed to reduce the pleiotropic effects derived from constitutive overexpression of early flowering genes. For instance, Sinn et al. (2021) developed transgenic grapefruits expressing *P. trifoliata* FT1 (PtFT1) as a translational fusion with a single-chain variable fragment antibody. The reduced FT activity rendered transgenic FT chimeras with precocious flowering.

The huge amount of genomic data available for citrus species and phylogenetically related genus would make the discovery of new genes involved in precocious flowering possible. This is the case for Mini-citrus (*Fortunella hindsii*), wild citrus-related species with dwarf height and early flowering (juvenile period of around 8 months) (Zhu et al., 2019). The discovery of new candidate genes for transgenic or gene editing approaches would speed up the implementation of biotechnological improvements in citrus species (Shimizu, 2020; Rao et al., 2021).

## Novel Strategies for Transient and Stable Transformation of Citrus

Generation of stably transformed citrus plants requires arduous and time-consuming procedures, as is the case for most woody species. As mentioned before, the success depends on genotype-associated transformation efficiencies and needs long periods for *in vitro* regeneration of positive events. Also, the principal characteristics of the candidate genes to introduce before conducting stable transformation of the plants should be previously analyzed. For that purpose, transient gene expression is a useful tool to study the function, subcellular expression patterns, and localization of the genes of interest. This method also allows characterizing novel genes and regulatory sequences in a fast and simple manner (Jones et al., 2009).

In citrus, the study of Figueiredo et al. (2011) has determined the function and subcellular localization of a type III effector AvrGf1 from *Xanthomonas citri pv. citri* by *Agrobacterium*-mediated transient expression (agroinfiltration) in grapefruit leaves. In addition, it has been suggested that a *X. citri pv. citri* treatment before agroinfiltration could significantly enhance transient expression in recalcitrant citrus leaves from different varieties (Jia and Wang, 2014b). The constraints for this pre-treatment are related to the possible side effects derived from the presence of pathogenic bacteria that could interfere with the functional characterization of the gene of interest. Li et al. (2017) have analyzed different factors influencing transient expression efficiency in citrus. They suggested composition of infiltration buffer adequate for an enhanced level of transient expression: 10 mmol L^–1^ 2-(N-morpholino) ethanesulfonic acid (MES), pH 5.6, 10 mmol L^–1^ MgCl_2_, and 150 μmol L^–1^ Acetosyringone. The bacterial suspension density most suitable for transient expression was OD_600_ = 0.8. The optimal conditions of other parameters, such as temperature, leaf developmental stages, and dependence on genotypes, were also determined. A recent study has shown that an agroinfiltration procedure using a microneedle roller to create abundant little wounds in the leaf surface increased the gene expression efficiency (Acanda et al., 2021). Strategies for transient gene expression based on particle bombardment have been also developed. A held-gene gun system was used for transient transformation of thin epicotyl explants of Carrizo citrange and sweet orange (Bespalhok Filho et al., 2003) as well as for citrus leaves (Levy et al., 2018). The latter system is applicable to a wide variety of genotypes but not all laboratories have the required device. Another novel methodology for transient citrus fruit transgene expression based on fruit immersion in an *Agrobacterium* suspension and subsequent vacuum infiltration has been reported by Zhang et al. (2021).

**FIGURE 4:**
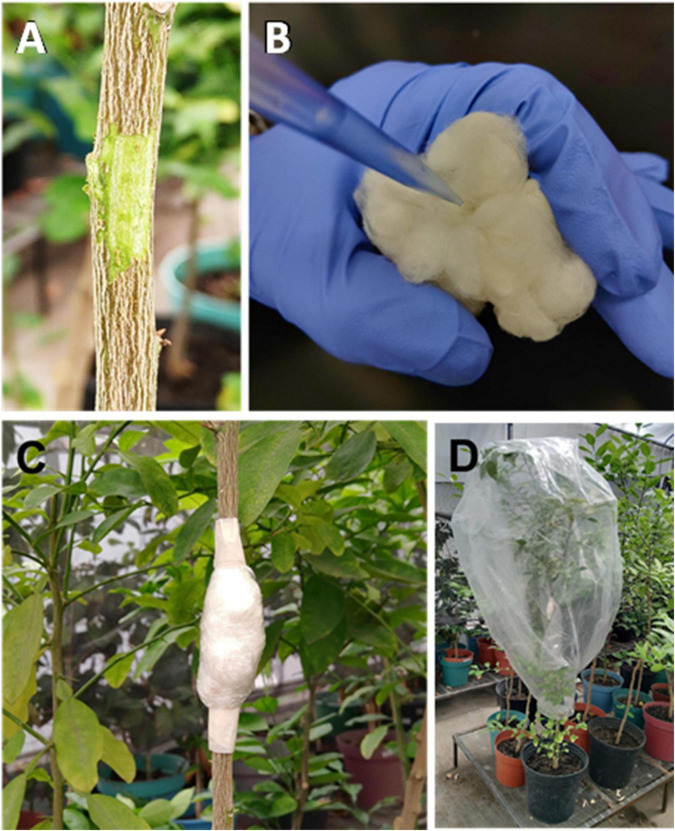
Localized expression procedure. **(A)** Exposition of photosynthetic tissue by scraping made with a scalpel. **(B)** Soaking of a cotton swab with *Agrobacterium* culture. **(C)** Wrapping of plant tissue. **(D)** Treated plant, covered with plastic.

A localized expression is a rapid tool allowing testing the correct expression of a transgene. In this regard, Guerra-Lupián et al. (2018) have developed a method for localized expression in stems of Mexican lime by using *A. tumefaciens* and expression vectors coding for reporter genes and antimicrobial peptides targeted to the vascular tissues (López-Buenfil et al., 2017; Guerra-Lupián et al., 2018). Stem transformation with *A. tumefaciens* carrying the transgene constructs requires a slight scraping of the corky surface with a scalpel, in order to expose the photosynthetic tissue, and then it is incubated with the bacteria to favor the transformation process (Figure 4). In some instances, the appearance of thick photosynthetic tissue is observed in the injury performed with the scalpel; in general, the scar is lignified and becomes indistinguishable over time (López-Buenfil et al., 2017). Molecular detection in the site of the transformation for evaluation of transgene expression can produce data that are difficult to interpret since *Agrobacterium* cells can be viable for weeks at the site of inoculation. However, it has been possible to detect reporter gene products systemically, in distant tissue. This fact is very interesting because informational molecules can be mobilized *via* phloem at long distances in the plant, without having to generate genetically modified plants.

The alternative methodology to achieve stable transgene expression by agroinoculation of axillary meristems is very useful to bypass the complexity and time-consuming procedures required for regenerating a whole plant from a single cell. He et al. (2011) have developed transgenic Jincheng orange and Newhall navel orange overexpressing antibacterial Shiva A and Cecropin B proteins by *Agrobacterium*-transformation of *in vitro* micrografted mature axillary buds. The transgenic plants subsequently regenerated showed resistance to *X. citri pv citri*.

An interesting new *in planta* transformation approach developed by Zhang Y. Y. et al. (2017) consists of an *Agrobacterium* co-culture with decapitated pummelo seedlings, selective-culture, and dark treatment for inducing bud formation. This strategy resulted in transgene integration with transformation efficiencies of 20.41% when using hygromycin as the selection marker and of 19.37 and 3.21% when using Basta and Kanamycin, respectively.

Other relevant tools for the expression or silencing of citrus genes are the viral-based expression vectors (Folimonov et al., 2007). Citrus Tristeza Virus (CTV)-based engineered constructs expressed the GFP reporter gene for more than 4 years (Folimonov et al., 2007). CTV showed stable expression of transgenes placed within the 3′ region of its genome (El-Mohtar and Dawson, 2014). Velázquez et al. (2016) have reported the use of CLBV for inducing early flowering of juvenile citrus plants, by transient expression of citrus Flowering Locus T gene (CiFT). This viral vector displayed a series of advantages, such as the absence of plant genome integration or recombination, the scarce range of symptoms expressed in most citrus cultivars, its systemic distribution in the plant, and its safety for field trials, because it is not transmissible by insects.

## Emerging Technologies: *Cis* and Intragenesis, *Trans*-Grafting and Gene Editing in Citrus

In citrus species, as well as in other woody fruit species, breeding programs based on transformation methods using *Agrobacterium* or biolistic strategies, have been successful in the precise insertion of foreign DNA for the improvement of desired traits without altering the genetic background. However, the presence of genes from other species, the use of selectable markers, and regulatory sequences coming from viruses or bacteria raised considerable public concerns. In this regard, a series of new breeding technologies (NBTs) was developed to modify existing DNA sequences in a plant or to modulate the patterns of endogenous gene expression and were successfully implemented for woody fruit plants in recent years (reviewed in Limera et al., 2017; Poles et al., 2020). *Cis* and intragenesis, *trans*-grafting, and gene editing techniques will be considered within this category. Although their application for citrus genetic improvement is still limited, some examples can be mentioned.

Cisgenic and intragenic plants are genetically modified organisms bearing DNA sequences from the species itself (extra copies) or from a closely related species that can be crossed conventionally, in contrast to transgenesis, where genetic material can be mixed between species. In the case of cisgenesis, the natural complete variant includes the promoter, introns, and terminator sequences in the same orientation as the native gene (Lusser and Davies, 2013). In intragenesis, the introduced DNA sequence can be a combination of genes and regulatory sequences (chimeric gene rearrangements) that will lead to different functional versions (Rommens et al., 2007). Recently, several citrus genes that control traits of interest have been cloned and characterized as novel targets for *cis* and intragenesis approaches. Between them CsSAMT1 (*Salicylic acid Carboxyl Methyltransferase 1*), which confers tolerance to HLB; CsMADS5, a fruit ripening-associated transcription factor, that positively regulates carotenoid biosynthesis in citrus; a CsMYB96 transcription factor, which enhances citrus fruit resistance against fungal pathogens; CiMADS43, a MADS-Box gene involved in citrus flowering and leaf development and CiNPR4, an NPR1-like gene that enhanced resistance of transgenic citrus plants to HLB (Li et al., 2020; Khan et al., 2021; Lu et al., 2021; Peng et al., 2021b; Zeng et al., 2021; Zhang et al., 2021; Zou et al., 2021).

*Cis* and intragenic citrus plants must be transformed with citrus-derived DNA sequences. For that purpose, in the case of the *Agrobacterium* transformation method, a suitable transformation vector system that carries citrus-derived complete T-DNA sequences is desirable. Plant-derived transfer DNA (P-DNA) was already developed to replace bacterial vector T-DNA backbone with a plant DNA sequence (Rommens, 2004). For citrus genetic transformation, An et al. (2013) have developed an intragenic vector system by adding a T-DNA-like sequence from *C. clementina*, in the correct orientation and with a series of restriction sites for cloning the gene of interest. The empty vector was used to transform *A. thaliana* and Duncan grapefruit leading to the recovery of positive events under non-selective conditions (3 and 0.67% transformation efficiencies for both species, respectively).

In citrus, the use of reporter genes based on the production of anthocyanin (Dutt et al., 2016, 2018b; Huang et al., 2019) and systems to remove selectable markers have shown positive results (Sugita et al., 1999; Zou et al., 2013). Merritt et al. (2021) developed a citrus DNA glyphosate resistant-selection system by transforming Duncan grapefruit with a glyphosate-resistant mutated version of citrus EPSPS enzyme (TIPS EPSPS). They showed that a glyphosate treatment did not inhibit bud formation and rendered a 40% increment in transformation efficiency. A third requirement is that the native target gene must be linked to a suitable regulatory sequence. For that purpose, several citrus-derived promoters are being characterized (Erpen et al., 2018; Dasgupta et al., 2020; Bezerra et al., 2021).

*Trans*-grafting is a relevant practice in citrus biotechnological breeding that combines genetic engineering with traditional grafting practices. This method consists of grafting a non-genetically modified scion onto a transgenic rootstock (Kaiser and Dalton, 2001; Haroldsen et al., 2012). Thus, in *trans*-grafted plants, mobile transgene products can move across the phloem from the transgenic rootstock to the non-transgenic scion, so that the latter can acquire the beneficial trait with no-genetic modification of the final products, for example as in fruits (Song et al., 2015). In citrus crops, non-transgenic scions susceptible to HLB bacterial disease were grafted onto transgenic rootstocks overexpressing a microbial peptide and the resulting plants showed lower rates of infection compared with non-transgrafted plants (Bergey et al., 2015). De Francesco et al. (2020) trans-grafted a transgenic sweet orange interstock overexpressing a hairpin CP-mRNA and observed tolerance to citrus psorosis virus in the non-transgenic scion.

New genome engineering technologies offer encouraging alternatives to create mutations in the citrus genome. For example, the recently developed clustered regularly interspaced short palindromic repeats (CRISPR)-associated Cas9 genome editing tool has been successfully applied to citrus species. The efficacy of the CRISPR/Cas9 technique in citrus plants was first studied by targeting the phytoene desaturase (PDS) gene (Jia and Wang, 2014a). The disruption of this gene impairs chlorophyll and carotenoid production resulting in albino or mosaic phenotypes that can be observed visually to estimate the efficacy of the genome modification system (Qin et al., 2007). The study of Jia and Wang (2014a) first reported PDS editing in *C. sinensis* plants, with a very low editing efficiency of about 3.5%. The work of Zhang F. et al. (2017) has lately developed a *higher* efficient CRISPR system to edit PDS that relies on (1) Cas9 driven by the Arabidopsis YAO-promoter instead of 35S, and (2) a bifunctional selectable marker used to identify transgenic citrus plants with high expression of Cas9. They obtained albino phenotypes consistent with high mutation frequencies of up to 75% (Zhang F. et al., 2017). The authors also reported that most of the mutations obtained in their study were identified as indels that resulted in a frameshift.

On the other hand, the study of Jia et al. (2017) recently reported editing of the CsLOB1 (*C. sinensis Lateral Organ Boundaries*) gene. CsLOB1 is a susceptibility gene for citrus canker disease and is induced by the pathogenicity factor PthA4 from *X. citri* pv. citri. PthA4 binds to the EBEPthA4-CsLOBP to induce CsLOB1 gene expression (Hu et al., 2014). CsLOB1 was targeted for edition both in its promoter and coding sequences, in Valencia sweet orange (*C. sinensis*) and Duncan grapefruit (*C. paradisi* Macf.) (Jia et al., 2016, 2017; Peng et al., 2017). The efficiency of recovering mutant plants spanned from 23 to 67% and the transgenic lines with higher mutation rates became resistant to citrus canker. In addition, the generation of homozygous and biallelic canker-resistant Pummelo (*C. maxima*) plants in the T0 generation was reported (Jia and Wang, 2020). CRISPR edition of CsWRKY22 gene, a marker gene for pathogen-triggered immunity in *C. sinensis* also reduces susceptibility to *X. citri* subsp. citri in Wanjincheng orange (Wang et al., 2019).

The LOB1 promoter was also edited in Duncan grapefruit using SaCas9 from *Staphylococcus aureus* (Jia et al., 2016) instead of the commonly used SpCas9 from *Streptococcus pyogenes*. SaCas9 (Ran et al., 2015) is a 1053 aa nuclease, much smaller than SpCas9 (1368 aa), that allows easier handling and target cell transformation. No off-targets were observed when using SaCas9 in citrus gene editing neither in transgenic edited tobacco (Kaya et al., 2016). CRISPR-Cas12a from Prevotella and Francisella (a class II/type V CRISPR nuclease), has been also employed to edit PDS or CsLOB1 genes in Duncan grapefruit (Jia et al., 2019) and has been proposed as an alternative system reported having fewer off-targets in relation to Cas9 (Kim et al., 2016; Kleinstiver et al., 2016). The work of Dutt et al. (2020) have recently reported the edition of the PDS gene in *C. sinensis* plants using embryogenic callus as explants instead of epicotyls. They employed two different constructs: one where the gRNAs were driven by the *Arabidopsis* U6–26 pol III promoter and another with the RNA processing ability of the Csy4 bacterial endoribonuclease to express several gRNAs (Čermák et al., 2017). All the generated transgenic embryos were completely albino, and no variegated phenotype was observed, which demonstrates a high editing efficiency (Dutt et al., 2020). The embryogenic cell culture mediated transformation system allows a larger population of transformed plants compared with the epicotyl explant mediated system. Furthermore, seedless citrus cultivars and epicotyl transformation recalcitrant cultivars can be easily transformed with this system.

## Perspectives

Citrus improvement requires a continuing effort for success. Emerging biotechnologies are providing the research community with new tools that can increase the speed and efficiency of the process. Public perception of transgenic citrus, however, is an actual concern and should be taken into consideration. Even though it could be argued that pathogen-resistant transgenic citrus could improve sustainability by reducing pesticide applications (Caserta et al., 2019), they nevertheless bring other concerns. Some of them are the effect of the modified plants on the environment, the risk of transgene dissemination by pollen, the potential damage to local production and small growers, who cannot adopt the new technology, and potential risks to human health due to the consumption of transgenic citrus fruits, among others.

The expression of genes from citrus origin to obtain cisgenic or intragenic varieties is a promising strategy, considering the issues around the public perception of transgenic plants and the need to address the reduction of the regulation (Holme et al., 2013). When the genetic sequences to be introduced originate in closely related species, instead of phylogenetically distant ones, regulatory processes become easier to achieve. Furthermore, the application of CRISPR/Cas technologies has the potential of generating non-transgenic-edited citrus plants, thus falling within a different regulatory framework (Lema, 2019). In many cases, the result of editing genetic sequences is comparable to that obtained by conventional mutagenesis, if there are no leftovers of Cas9 and gRNA (guide RNA) sequences inserted in the genome. To move toward those alternatives, it is necessary to modify current transformation methods for citrus editing. Promising strategies may include the use of Cas9-ribonucleoprotein complexes for the transitory transformation of either protoplast with PEG or callus explants by biolistic, eluding transgene integration in the plant genome. Therefore, the edited plant can be classified as non-transgenic according to the Cartagena Protocol on Biosafety (Lema, 2019).

## Author Contributions

GC and CAR: conceptualization, investigation, supervision, and writing—original draft. BX-C and GM-P: writing—original draft and funding acquisition. HEH: supervision and funding acquisition. All authors contributed to the article and approved the submitted version.

## Conflict of Interest

The authors declare that the research was conducted in the absence of any commercial or financial relationships that could be construed as a potential conflict of interest.

## Publisher’s Note

All claims expressed in this article are solely those of the authors and do not necessarily represent those of their affiliated organizations, or those of the publisher, the editors and the reviewers. Any product that may be evaluated in this article, or claim that may be made by its manufacturer, is not guaranteed or endorsed by the publisher.

## Footnotes

1
www.fao.org

2
https://knoema.com/
